# Changes in the quality of medicines during storage under LED lighting and consideration of countermeasures

**DOI:** 10.1186/s40780-018-0108-0

**Published:** 2018-06-01

**Authors:** Shuuji Yamashita, Kazuhiro Iguchi, Yoshihiro Noguchi, Chihiro Sakai, Satoshi Yokoyama, Yoko Ino, Hideki Hayashi, Hitomi Teramachi, Magoichi Sako, Tadashi Sugiyama

**Affiliations:** 1 Laboratory of Pharmacy Practice and Social Science, Gifu Pharmaceutical University, 1-25-4 Daigaku-nishi, Gifu, Gifu 501-1196 Japan; 2 Gifu Pharmaceutical University Pharmacy, 1-108-3 Daigaku-nishi, Gifu, Gifu 501-1113 Japan; 3 Laboratory of Community Pharmacy, Gifu Pharmaceutical University, 1-25-4 Daigaku-nishi, Gifu, Gifu 501-1196 Japan; 4 Laboratory of Clinical Pharmacy, Gifu Pharmaceutical University, 1-25-4 Daigaku-nishi, Gifu, Gifu 501-1196 Japan; 5 Department of Community Healthcare Pharmacy, Gifu Pharmaceutical University, 1-25-4 Daigaku-nishi, Gifu, Gifu 501-1196 Japan; 6 Research and Development Department, Ohara Pharmaceutical Co. Ltd., 121-15 Toriino, Koka-cho, Koka-shi, Shiga 520-3403 Japan

**Keywords:** LED lighting, Fluorescent lighting, Photostability, Color change, Medicines

## Abstract

**Background:**

In recent years, the popularity of LED lighting has rapidly increased, owing to its many advantages, including economic benefits. We examined the change in the quality of drugs during storage under LED and fluorescent lighting and found that some medicines exhibited a different degree of color change depending on the light source. The purpose of this study was to investigate the effects of different plastic storage bags on the color change over time when various medicines were stored under LED and fluorescent lighting conditions.

**Methods:**

Photostability tests were conducted on several types of target drugs. Subsequently, subjective evaluation by ten evaluators and objective evaluation by image analysis software were carried out regarding color change.

**Results:**

A similar change in color tone was observed after all types of illumination. Subjective evaluation by 10 evaluators revealed that “change in color tone” occurred in the order of bulb-color LED lighting < daylight-color LED lighting < fluorescent lighting, regardless of the type of plastic bags. A similar tendency was observed also in objective evaluation. In this study, it was considered that a brown light-shielding plastic bag was more effective than a normal plastic bag for the prevention of the color change of medicines stored under LED lighting.

**Conclusions:**

The above results suggested that the most appropriate combination of plastic bag and light source for medicine storage was a brown light-shielding plastic bag and bulb-color LED lighting.

**Electronic supplementary material:**

The online version of this article (10.1186/s40780-018-0108-0) contains supplementary material, which is available to authorized users.

## Background

Medicines are affected by external factors, such as temperature, humidity, and light, during storage in medical institutions and patient homes. These cause physical and chemical changes; a change in appearance and a decrease in titer have been reported [1–3]. For this reason, according to the test results based on the “Guidelines for Photostability Test of New Media and New Products” [4], pharmacists agreed on the need to maintain a consistent storage environment for medicines in the dispensing room, ensure the quality of medicines, and to instruct patients about appropriate storage methods at the “International Conference on Harmonization of Technical Requirements for Registration of Pharmaceuticals for Human Use”.

Sometimes medicines are stored in a plastic bag at the dispensing room of a medical institution or the patient’s home. Polybags are made of various materials, but in general they often refer to those made of polyethylene. In addition, various additives are likely added in the manufacturing process in order to impart various functions such as light shielding, antistatic and antimicrobial.

Fluorescent lighting is a light source expected to be widely encountered in the dispensing room of a medical institution or a patient’s home. However, owing to the advantages of energy conservation, such as less power consumption and lower heat generation, and the reduction of the burdens on the natural environment, such as long energy-saving period and low emission of ultraviolet light, LED lighting has been increasingly used in both ordinary households and medical institutions. The light source color of a bulb-type LED lamp is divided into five kinds of daylight color, day white color, white color, warm white color and light bulb color based on Japanese Industrial Standards [5]. The difference in light source color depends on the difference in correlated color temperature (K), which affects not only the impression given to the room but also the energy consumption efficiency.

We examined the differences in the degree of the color change of various medicines after exposure to LED and fluorescent lighting. A noticeable degree of color change was observed for the Lasix® 20 mg Tab. and Parlodel® 2.5 mg Tab. [6, 7]. However, the differences in the stability of medicines stored in different plastic bag types under LED lighting have not been examined so far. Therefore, the purpose of this study was to assess the effect of different plastic bag types on the color change of medicines over time after storage under LED lighting and fluorescent lighting.

## Methods

### Pharmaceuticals

The target drugs were furosemide (Sanofi KK, Lasix® 20 mg Tab., Tokyo, Japan), bromocriptine mesylate (Sun Pharmaceutical Industries Ltd., Parlodel® 2.5 mg Tab., Mumbai, India), trichlormethiazide (Shionogi & Co, Fluitran® 2 mg Tab., Osaka, Japan), mequitazine (Alfresa Pharma Corp., Nipolazin® 3 mg Tab., Osaka, Japan), and paracetamol (Ayumi Pharmaceutical Corp., Ltd., Calonal® 200 mg Tab., Tokyo, Japan), which represented five types of medicine.

### Lighting conditions and LED meter

The lighting conditions were daylight-color LED bulb (Panasonic Corp., LDA 11 DG, Tokyo, Japan), bulb-color LED (Panasonic Corp., LDA 10 LG/Z 60 W, Tokyo, Japan), and bulb type fluorescent lighting (Panasonic Corp., EFA 15 EN 10 H 2, Tokyo, Japan). The color temperature of each light was 6700 K, 2700 K, and 5000 K, respectively. The wavelength spectrum of each lighting was measured with an LED meter (UPRtek Corp., MK 350, Miaoli, Taiwan).

### Polybag with seal

**Fig. 1:**
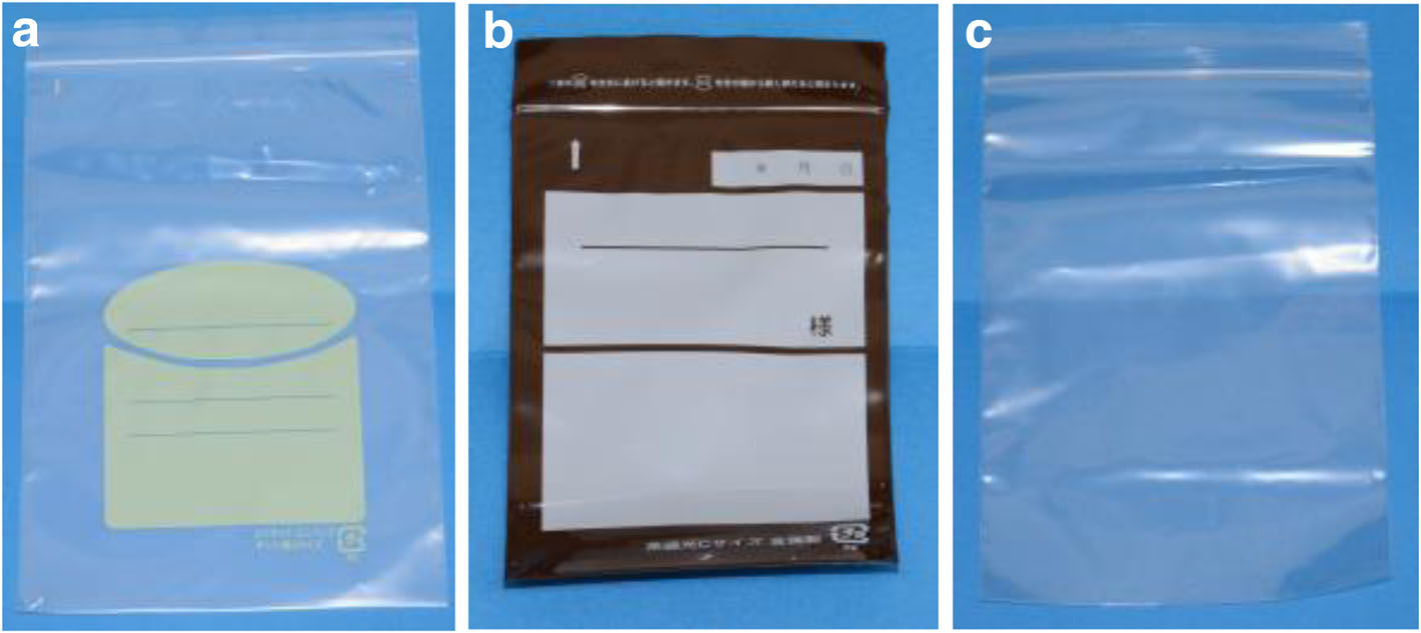
Pictures of various polybag with seal in this study. UV-cut plastic bags (**a**), brown light-shielding plastic bags (**b**), and normal plastic bags (**c**)

When storing the target drugs, UV-cut plastic bags (Kinshi Seisakujo Co., Ltd., 200 mm × 140 mm (G-5), Tokyo, Japan), brown light-shielding plastic bags (Kinshi Seisakujo Co., Ltd., 100 mm × 70 mm (C), Tokyo, Japan), and normal plastic bags (SHIMOJIMA Co., Ltd., SWAN Chakkupori (120 mm × 170 mm (F-4)), Tokyo, Japan) were used (Fig. 1).

### Test of photostability of target medicines and evaluation

A total of five medicines (Lasix®20 mg Tab., Parlodel® 2.5 mg Tab., Fluitran® 2 mg Tab., Nipolazin® 3 mg Tab., and Calonal® 200 mg Tab.) were placed in several types of plastic bags with a seal (UV-cut function; brown with light-shielding function; normal without light-shielding function) and exposed for up to 28 days (approximately 670,000 lx·h) to daylight-color LED lighting, bulb-color LED lighting, fluorescent lighting, and in the dark.

The color change of the exposed medicine was evaluated subjectively by the evaluators, who were 10 practical interns accepted at this facility, between May 9 and July 22, 2016. They were students of the 22–25 year old pharmaceutical department, 4 men and 6 females. We instructed the evaluators to observe the medicines after different exposure periods. Thereafter, the evaluators reported whether there was a color change between the medicine exposed to the different illumination conditions and the medicine left in the dark. The target medicine was described as “change in color tone” or “no change in color tone”.

Changes in color tone of the target drug after the test of photostability were analyzed using image analysis software ImageJ (version 1.51, National Institutes of Health, USA). The mean gray value of the relevant part of the image (Additional file 1) obtained by photographing the object drug after the test of photostability was measured. In addition, mean gray value was measured for each medicine at three places, and the average value of the mean gray value calculated respectively was used.

## Results

### Measurement result of wavelength spectrum

The white LED employs the blue LED and the yellow phosphor to obtain white light, so the peak appeared in two places. Bulb color and daylight color were the same LED lighting, but differences in spectrum were seen due to different light colors. On the other hand, the white fluorescent lamp had many peaks due to mercury emission line accompanied by discharge and light obtained by synthesizing the emission spectrum of the phosphor.

### Changes in the quality of medicines under each condition

Additional file 1 shows the results of exposing each medicine at 1000 lx for up to 28 days under LED and fluorescent lighting. During different exposure conditions, the temperature was maintained at 24.8 ± 4 °C and the humidity was 56 ± 16%.

**Fig. 2:**
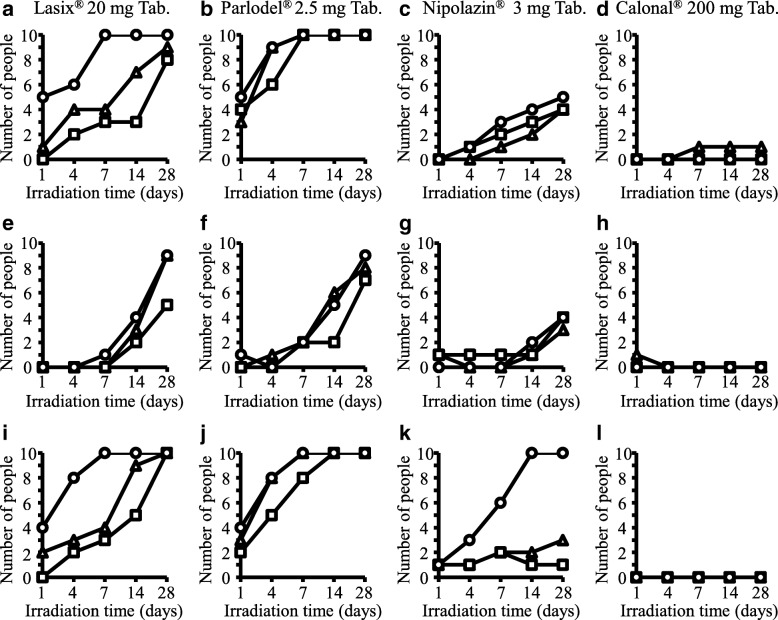
Subjective evaluation of the color change of various medicines. Indicates the number of evaluators who evaluated “change in color tone” for each medicine after the test of photostability. The color change of various medicines was evaluated after storage for 1 day to 28 days in three lighting conditions (Daylight-color LED lighting (△), bulb-color LED lighting (□) and fluorescent lighting (○)) in UV-cut plastic bags (**a**-**d**), brown light-shielding plastic bags (**e**-**h**), and normal plastic bags (**i**-**l**)

Figure 2 shows the total number of evaluators evaluated as “change in color tone”. After 7 days, all 10 evaluators reported “change in color tone” for Lasix®20 mg Tab., Parlodel® 2.5 mg Tab., and Fluitran® 2 mg Tab. stored in UV-cut polybags or normal plastic bags without a light-shielding function under fluorescent light (Fig. 2a, b, i, and j). Similar color tone changes were observed under all illumination conditions. After 14 days, all 10 evaluators reported “change in color tone” for Nipolazin® 3 mg Tab. in normal plastic bags stored under fluorescent lighting (Fig. 2k). After 28 days, five evaluators indicated a “change in color tone” for Nipolazin® 3 mg Tab. stored in UV-cut plastic bags and four evaluators indicated this for the Nipolazin® 3 mg Tab. stored in brown light-shielding plastic bag under fluorescent lighting (Fig. 2c, g). The number of evaluators who reported “change in color tone” showed the following trend: bulb-color LED lighting < daylight-color LED lighting < fluorescent lighting (Fig. 2). For brown light-shielding plastic bags, the number of evaluators who reported “change in color tone” tended to be smaller for the target medicines in comparison with that observed for the other types of plastic bags (Fig. 2). For Calonal® 200 mg Tab., almost no changes in color tone were observed under any storage conditions (Fig. 2d, h and l).

**Fig. 3:**
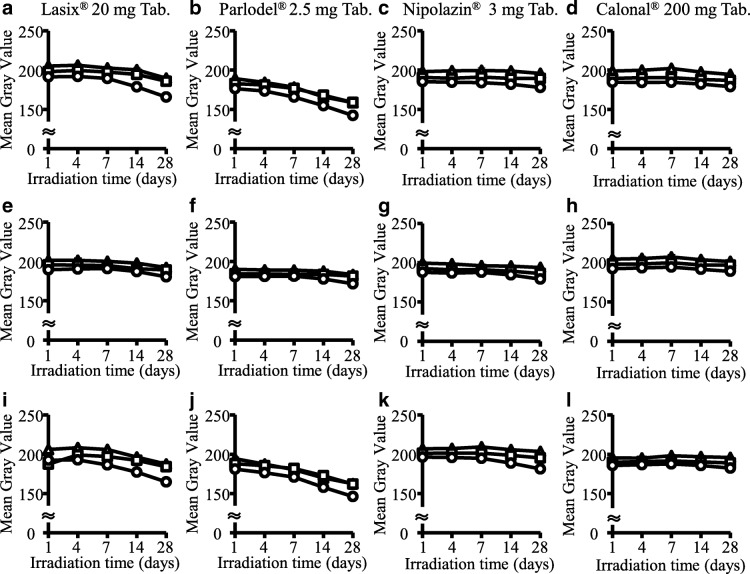
Objective evaluation of color change of various medicines. The results of objectively evaluating the color change of various medicines after the test of photostability was shown. Mean gray value was estimated using ImageJ. The color change of various medicines was evaluated after storage for 1 day to 28 days in three lighting conditions (Daylight-color LED lighting (△), bulb-color LED lighting (□) and fluorescent lighting (○)) in UV-cut plastic bags (**a**-**d**), brown light-shielding plastic bags(**e**-**h**), and normal plastic bags (**i**-**l**)

Figure 3 shows the results of evaluating the color tone change after the test of photostability of each pharmaceutical product using ImageJ software. In Lasix®20 mg Tab., Parlodel® 2.5 mg Tab., and Nipolazin® 3 mg Tab., the mean gray value decreased under any light source, and a change in color tone was time dependent (Fig. 3). The condition that caused the largest color tone change was that the mean gray value was reduced by 34.9 when Parlodel® 2.5 mg Tab. was normal plastic bags under fluorescent lighting (Fig. 3j).

## Discussion

In this study, color changes were observed for many of the target medicines stored under LED lighting in both normal plastic bags and UV-cut plastic bags (Fig. 2a-d, i-l). However, when the target medicines were placed in brown light-shielding plastic bags, a smaller degree of change in the color tone was observed, suggesting they could be more effective than normal plastic bags (Fig. 2). Therefore, it was indicated that, depending on the conditions, shielding by UV-cut plastic bags may be insufficient to prevent the color changes that result from LED lighting (Fig. 2).

The color change was slower than in other light sources when stored under bulb-color LED lighting, regardless of the target medicine or the plastic bags used in this study (Fig. 2). Moreover, the gentlest light source for medicine was thought to be bulb-color LED lighting; this agreed with our previous reports [6, 7].

Lasix®20 mg Tab. showed almost no color change when exposed to light in the wavelength region of 420 nm or more, but was reported to undergo a remarkable color change after irradiation with light in the wavelength region below 420 nm [8]. In general, it is known that the photolytic degradation of medicines is wavelength-dependent; when the absorbed light energy is larger than binding energy of the substance, photolytic degradation occurs. If the decomposition product is colored, the color change can be visually observed. It is known that light of different wavelengths is easily absorbed by medicines owing to their differences in structure; even for identical illuminance, irradiation with light sources of different spectra produces different effects on medicines [9, 10]. In general, a white LED emits white light through a combination of a blue LED element and a yellow phosphor; therefore, the peak appears in two places. The difference in the emission spectrum of each type was attributed to the difference in the ratio of the peak of the blue emission spectrum of the element and the yellow emission spectrum of the phosphor. As shown in Figs. 2a, e and i, the degree of color change in Lasix®20 mg Tab. stored under the bulb-color LED lighting, in any type of bag, was the smallest. One explanation for this was that the total energy of 420 nm or less of the bulb-color LED lighting was lower than that of other LED lighting.

It is known that Parlodel® 2.5 mg Tab. showed a change in color tone after light irradiation [11]. The degree of color change in bulb-color LED lighting conditions (Fig. 2b, f and Additional file 1) was the smallest, even in Parlodel® 2.5 mg Tab., regardless of the type of plastic bags. Although the cause was unknown, reference to the past reports [11] and the measurement of the wavelength spectrum indicated that photosensitivity occurred over a small wavelength range, like that for Lasix®20 mg Tab.

It was been reported that Nipolazin® 3 mg Tab. absorbed light in the wavelength range 300–350 nm [12]. When Nipolazin® 3 mg Tab. was stored for 28 days in normal plastic bags, a distinct color change was observed under fluorescent lighting as compared to that with LED lighting. In the UV-cut plastic bag and the brown light-shielding plastic bag, there was no significant difference in the degree of color change among the lighting conditions. One explanation for this was that the relative emission intensity in the ultraviolet region, where Nipolazin® 3 mg Tab. mainly absorbs light, was lower than that of fluorescent lighting and the light-shielding performance in this wavelength region of the UV-cut plastic bag and the brown light-shielding plastic bag was sufficient in LED lighting.

The same tendency as the result of subjective evaluation was also observed in objective evaluation using ImageJ. Regarding Nipolazin® 3 mg Tab., the difference in each light source was not as clear as the result of subjective evaluation. Even taking this into consideration, we believed that the results obtained by objective evaluation support the results obtained by subjective evaluation.

The reason for choosing five drugs in this study was as follows: two drugs that were reported to exhibit a color change by the patient after dispensing and delivery at our facility (Lasix® 20 mg Tab. and Fluitran® 2 mg Tab.), medicines listed as having a color change in the interview form (Parlodel® 2.5 mg Tab. and Nipolazin® 3 mg Tab.), as a control for these, and a white medicine not described to exhibit a change in color tone (Calonal® 200 mg Tab.).

The degree of color change tended to become small when stored in brown light-shielding plastic bags for all combinations of medicines and light sources used in this study. We reported that subjective tendency to feel a sense of resistance to medication tended to be felt so that a change in color tone was felt [6]. Regardless of the decomposition of the principal component, changes in the appearance of medicines were easy to understand for the patient, and it was thought that it affected compliance as well. For this reason, we focused attention on the subjective color change of the evaluator and examined it from the PTP which seemed to be easier to understand color change than the PTP state. However, the effect on safety and efficacy owing to changes in the principal component and additives was also considered to be an important item to be evaluated. Therefore, we intend to quantitatively and qualitatively examine the changes in the principal component and additives of each subject drug and establish evidence for the proper storage of medicinal products.

## Conclusion

The above results suggested that the most appropriate combination of the plastic bag and light source for medicine storage was brown light-shielding plastic bags and bulb-color LED lighting.

## Additional file

Additional file 1: **Figure S1.** The color change of various medicines for 1 day to 28 days in three lighting conditions in UV-cut plastic bag (A), brown light-shielding plastic bag (B),normal plastic bags (C). (PPTX 23261 kb)

## Abbreviations

LED: Light emitting diode
PTP: Press through package
UV: Ultra violet
