# Supplementary material for: Changes in the quality of medicines during storage under LED lighting and consideration of countermeasures
Source: J Pharm Health Care Sci. 2018 Jun 1;4:12. doi: 10.1186/s40780-018-0108-0 (PMC5984437; doi:10.1186/s40780-018-0108-0)
Supplement: Supplementary file 1 — Figure S1. The color change of various medicines for 1 day to 28 days in three lighting conditions in UV-cut plastic bag (A), brown light-shielding plastic bag (B),normal plastic bags (C). (PPTX 23261 kb) [file 40780_2018_108_MOESM1_ESM.pptx]

## Slide 1
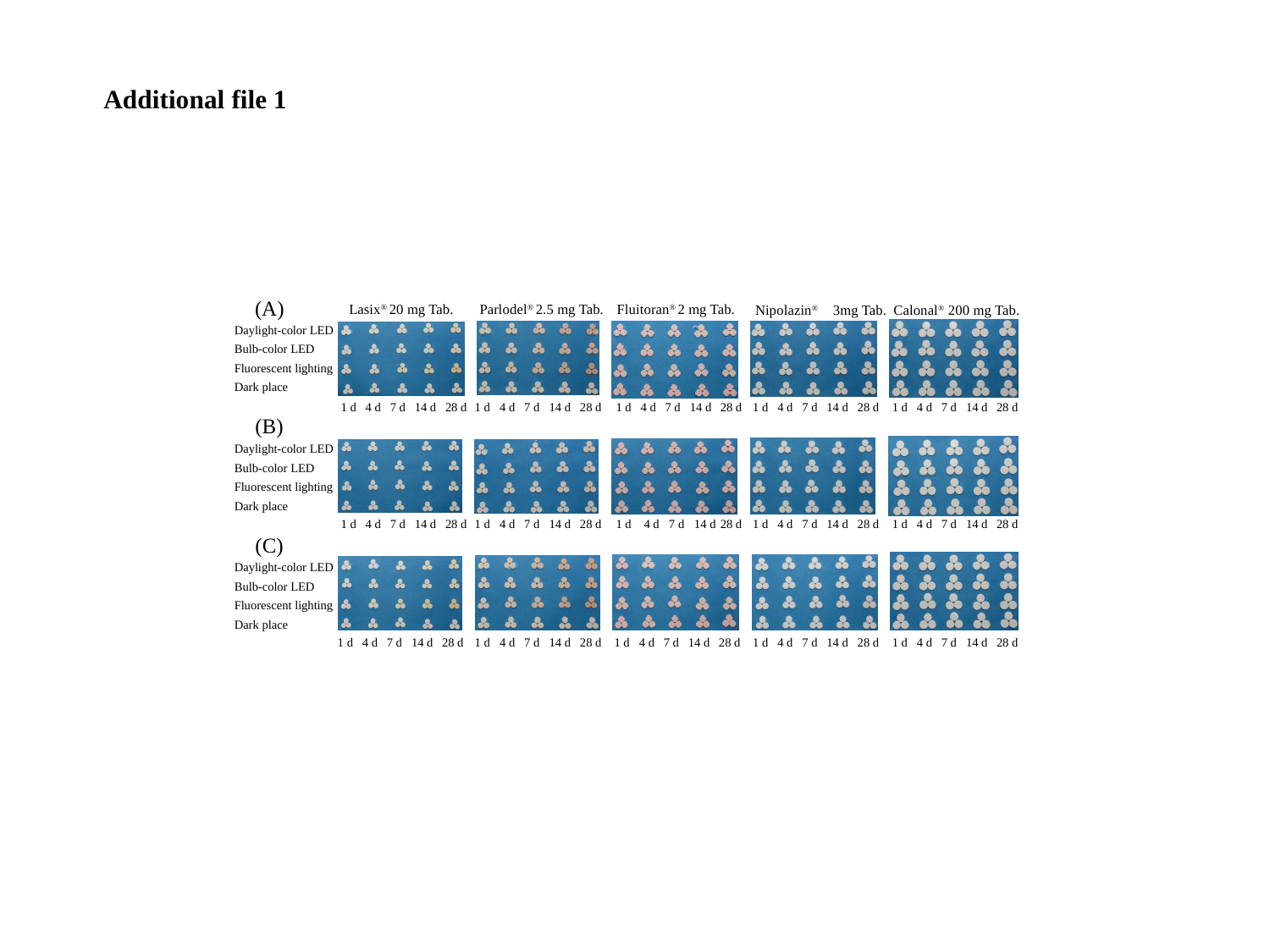

Additional file 1
(A)
Lasix® 20 mg Tab.
Parlodel® 2.5 mg Tab.
Fluitoran® 2 mg Tab.
Nipolazin®　3mg Tab.
Calonal® 200 mg Tab.
Daylight-color LED
Bulb-color LED
Fluorescent lighting
Dark place
1 d
4 d
7 d
14 d
28 d
1 d
4 d
7 d
14 d
28 d
1 d
4 d
7 d
14 d
28 d
1 d
4 d
7 d
14 d
28 d
1 d
4 d
7 d
14 d
28 d
(B)
Daylight-color LED
Bulb-color LED
Fluorescent lighting
Dark place
1 d
4 d
7 d
14 d
28 d
1 d
4 d
7 d
14 d
28 d
1 d
4 d
7 d
14 d
28 d
1 d
4 d
7 d
14 d
28 d
1 d
4 d
7 d
14 d
28 d
(C)
Daylight-color LED
Bulb-color LED
Fluorescent lighting
Dark place
1 d
4 d
7 d
14 d
28 d
1 d
4 d
7 d
14 d
28 d
1 d
4 d
7 d
14 d
28 d
1 d
4 d
7 d
14 d
28 d
1 d
4 d
7 d
14 d
28 d
